# The immunoregulatory role of helper T cells in *Helicobacter pylori* infection

**DOI:** 10.3389/fimmu.2025.1593727

**Published:** 2025-06-09

**Authors:** Xianli Wu, Guoyou Gou, Min Wen, Fang Wang, Youjia Liu, Lingli Li, Jingyu Xu, Rui Xie

**Affiliations:** ^1^ The Collaborative Innovation Center of Tissue Damage Repair and Regeneration Medicine of Zunyi Medical University, Zunyi, Guizhou, China; ^2^ Guizhou Provincial Key Laboratory for Digestive System Diseases, the Affiliated Hospital of Guizhou Medical University, Guiyang, Guizhou, China; ^3^ Department of Endoscopy and Digestive System, Guizhou Provincial People’s Hospital, Guiyang, Guizhou, China

**Keywords:** helper T cells, *Helicobacter pylori*, immunoregulation, cytokines, immune evasion

## Abstract

*Helicobacter pylori* (*H. pylori*) is a pathogenic bacterium that can survive in the human gastric mucosa and cause chronic infections. More than half of the global population is affected by *H. pylori* infection, which is closely associated with the development of gastritis, gastric ulcers, gastric cancer, and mucosa - associated lymphoid tissue lymphoma. The immune response triggered by *H. pylori* infection is complex and involves multiple immune cells. Among them, helper T cells (Th cells) play a crucial role in regulating the immune response. In recent years, researchers have conducted in - depth explorations of the immunoregulatory mechanisms, pathological effects of Th cells in *H. pylori* infection, and their applications in treatment. However, there are still problems such as insufficient understanding of the functions of Th cell subsets and unclear clinical treatment strategies. This paper aims to review the immunoregulatory role of Th cells in *H. pylori* infection, analyze their mechanism of action in the pathological process, and explore the future research directions and the development of treatment strategies, providing a theoretical basis for research in this field.

## 1 Introduction

*Helicobacter pylori* (*H. pylori*) is a Gram - negative bacterium that primarily colonizes the human gastric mucosa. It was first isolated in 1982 by Australian scientists Barry Marshall and Robin Warren (1). Since then, *H. pylori* has rapidly attracted extensive attention from the global medical community. This bacterium mainly inhabits the mucosal layer of the human stomach. Thanks to its unique biological characteristics, it can effectively evade the attack of gastric acid and survive in an acidic environment. By virtue of its specific colonization site, *H. pylori* minimizes the recognition of the innate immune system and thus escapes phagocytosis by the host. The global burden of *H. pylori* infection is extremely heavy, with a global prevalence of approximately 43.1%. The infection rate is even higher in developing countries, reaching over 70% (2). More seriously, *H. pylori* infection is one of the major risk factors for gastric cancer. In 1994, *H. pylori* was classified as a Group 1 carcinogen by the World Health Organization (WHO) (3–5). In recent years, research has shown that the immune system plays a crucial role in the occurrence and development of *H. pylori* infection. Helper T cells (Th cells), as key cells in the immune system, are responsible for regulating and enhancing the immune response.

As a core regulator of adaptive immune responses, the imbalance of Th cell subsets plays a crucial role in the pathogenesis of Hp infection. Early studies have revealed that the Th1/Th2 immune imbalance induced by Hp infection is a key factor driving the pathological process. Th1 cells primarily mediate cellular immunity, promoting the infiltration of neutrophils and macrophages in the gastric mucosa and triggering chronic inflammation. In contrast, Th2 cells rely on IL-4 and IL-13 to activate humoral immunity, and their excessive activation is associated with gastric mucosal barrier damage and allergic reactions (6). In recent years, the roles of Th17 cells and regulatory T cells (Tregs) have gradually been unveiled. Th17 cells promote the recruitment of neutrophils in the mucosal lamina propria and the expression of antimicrobial peptides by epithelial cells through IL-17, thereby participating in early defense. In contrast, Tregs inhibit excessive immune responses by secreting IL-10 and TGF-β. However, their abnormal activation may lead to impaired pathogen clearance and facilitate immune evasion (7). Although there is already a certain understanding of the immunoregulatory role of Th cells in *H. pylori* infection, the interactions among Th cell subsets and their complex regulatory mechanisms still need further in-depth study. For instance, the specific mechanisms by which Th1 and Th2, Th17 and Tregs are involved in *H. pylori* infection, as well as the regulatory mechanisms between them, remain unclear (7). Moreover, there are still significant knowledge gaps regarding the immune evasion mechanisms of *H. pylori* after infection and the regulatory network of the host immune response, especially how the dynamic interactions between Th cell subsets determine the infection outcome (clearance, persistence, or carcinogenesis) has not been fully elucidated. Therefore, a deep understanding of the immunoregulatory role of helper T cells in *H. pylori* infection and an analysis of how Th cells respond to *H. pylori* infection through different subset differentiations are of great significance for the prevention and treatment of *H. pylori* related diseases.

## 2 Pathophysiological mechanisms of *H. pylori* infection

*H. pylori* infection is a global health concern, affecting nearly half of the world’s population (8). It is the primary cause of chronic gastritis and peptic ulcers and is closely linked to severe diseases like gastric cancer and mucosa-associated lymphoid tissue lymphoma (9, 10). The pathogenic mechanism of *H. pylori* is complex, involving the interaction of bacterial biological characteristics, bacterial virulence factors, host immune responses, and environmental factors.

**Figure 1:**
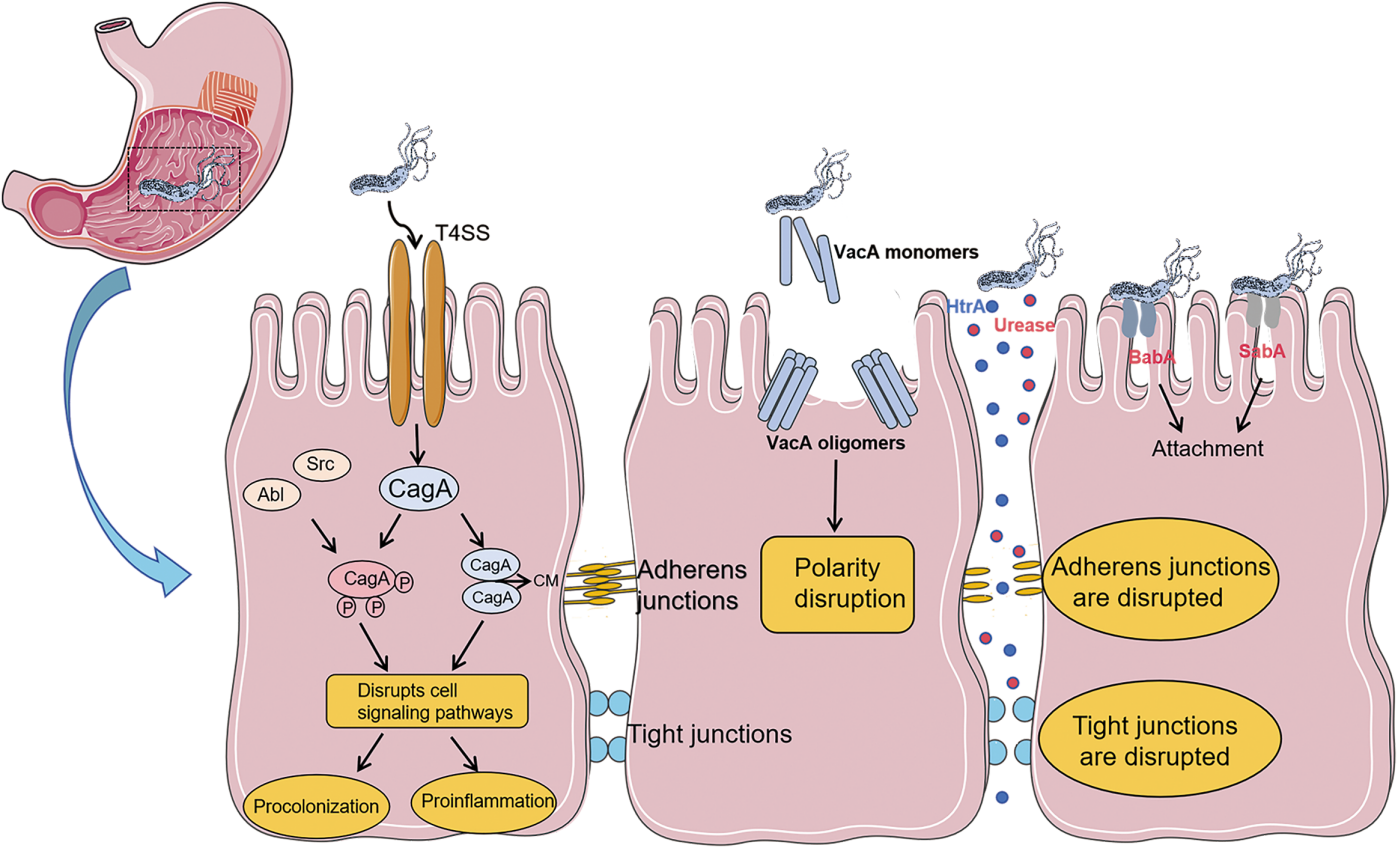
Pathogenic mechanism of virulence factors related to *Helicobacter pylori*: After *Helicobacter pylori* enters the gastric mucosa, the flagellar structure of *H. pylori* enables it to move in the mucus layer and penetrate deep into the submucosa of the gastric mucosa. It firmly adheres to gastric epithelial cells through adhesion factors (BabA, SabA). The urease secreted by Hp and the virulence factor HtrA directly disrupt cell junctions, damage the cell barrier, and promote bacterial attachment and persistence. The secretion of the virulence factor VacA will disrupt cell polarity. The virulence factor CagA is delivered into cells through the type IV secretion system. CagA can be phosphorylated by Src and Abl family kinases at the EPIYA motif located near the C-terminus. CagA can also form dimers in a phosphorylation-independent manner through the CagA polymerization (CM) sequence, and exerts its effects through both phosphorylation-dependent and phosphorylation-independent ways.

*H. pylori* can survive in the acidic environment of the stomach, mainly relying on its unique biological characteristics. *H. pylori* secretes urease, which can decompose urea in the stomach into ammonia, thereby neutralizing gastric acid and creating a micro- environment conducive to the survival of the bacteria. In addition, the flagellar structure of *H. pylori* enables it to move through the mucus layer, penetrate deep into the submucosa of the gastric mucosa, and firmly adhere to gastric epithelial cells through adhesion factors. This prevents the bacteria from being washed out of the gastric cavity by gastric juice, promotes bacterial colonization, and triggers a local inflammatory response (11). Research has shown that *H. pylori* can trigger an inflammatory response in host cells through its specific virulence factors. For example, the cytotoxin-associated vacuolating protein (VacA) can induce apoptosis of host cells, disrupt the epithelial cell barrier, and promote the inflammatory response. The cytotoxin - associated gene A (CagA) is translocated into the cytoplasm of host gastric epithelial cells through the *Helicobacter pylori* type-IV secretion system (12, 13). It is phosphorylated by host kinases at the Glutamic acid-Proline- Isoleucine-Tyrosine-Alanine (EPIYA) motif, activating multiple signaling pathways and leading to the production of pro - inflammatory cytokines and chemokines (14, 15). At the same time, it also causes cell proliferation, apoptosis, and genetic instability, significantly increasing the risk of gastric cancer (16). High-temperature requirement A (HtrA) can promote the infiltration and adhesion of inflammatory cells, exacerbating the severity of the inflammatory response (17, 18) (**Figure 1**).

In addition, the host’s immune response also plays a crucial role in *H. pylori* infection (19). *H. pylori* infection of the gastric mucosa triggers innate host defense mechanisms, including Nod1, which stimulates the gastric epithelial cells to express pro - inflammatory and antibacterial factors, leading to gastritis (20–23). Meanwhile, during *H. pylori* infection, innate immune cells such as neutrophils, monocytes, macrophages and dendritic cells (Dendritic cells, DCs) are activated. These cells initiate an immune response by recognizing characteristic bacterial molecules (such as lipopolysaccharide and flagella), releasing cytokines (such as tumor necrosis factor-α and interleukin-8), and attracting more immune cells to the site of infection to exert antibacterial effects. However, these inflammatory cells can produce and release inflammatory mediators, including reactive oxygen species, which cause oxidative stress in the adjacent gastric epithelial cells, affect gene expression, and induce DNA damage, resulting in a strong inflammatory response. Instead of clearing the bacteria, this can cause damage to the gastric mucosa. Gastric mucosal damage caused by inflammation can lead to gastric mucosal atrophy, intestinal metaplasia, and mutations in genes related to the development of gastric cancer (24). Secondly, the adaptive immune response also plays an important role in *H. pylori* infection. B cells and T cells can generate specific antibodies and cell - mediated immune responses by recognizing specific antigens. Research shows that the antibodies produced after *H. pylori* infection are mainly IgG and IgA, which can, to some extent, neutralize the bacteria and their toxins. However, *H. pylori* has various mechanisms to evade immune surveillance (25). *H. pylori* can modulate T cell responses to achieve immune evasion through its virulence factors CagA and VacA. After CagA is injected into gastric epithelial cells via the type IV secretion system, it not only activates oncogenic signaling pathways but also induces the expression of programmed death ligand 1 (PD-L1) in epithelial cells, directly inhibiting the cytotoxic function of CD8+ T cells. Meanwhile, CagA can also activate the STAT3 signaling pathway, promoting the differentiation of Tregs and creating an immunosuppressive microenvironment (26). VacA, on the other hand, induces T cell apoptosis by disrupting mitochondrial function and selectively inhibits Th1 cell secretion of IFN-γ, while promoting Th2 cell differentiation, thereby shifting the Th1/Th2 balance towards immune tolerance (27). In addition, the antigenic variability of *H. pylori* and the chronic immune activation loop are also important mechanisms for its immune evasion. The surface antigens of *H. pylori* (such as BabA/B, SabA) are highly variable, which can evade specific recognition by T cells, leading to immune evasion (28, 29). The persistent presence of *H. pylori* continuously stimulates the immune system, resulting in chronic immune activation. This, in turn, leads to chronic gastritis, dysfunction of immune cells in the gastric mucosa, and further promotes the development of gastric diseases (25).

Previous studies have shown that mouse models have played a key role in elucidating the pathogenic mechanisms of *H. pylori*. However, there are significant differences between mice and humans in terms of the gastric environment, immune system responses, and the interactions between bacteria and the host. For instance, the gastric pH of mice is significantly higher than that of humans, which makes it difficult to fully reproduce the characteristics of human chronic gastritis in terms of bacterial colonization density and inflammatory phenotypes. Moreover, mice lack the receptors for *H. pylori* virulence factors that are unique to humans, resulting in deviations in T cell polarization patterns from clinical observations. To overcome the limitations of animal models, human organoid-immune co-culture systems are becoming a research hotspot (30). This system mimics the mucosal microenvironment through gastric organoids, including gastric epithelial cells, immune cells, and the extracellular matrix, allowing for real-time monitoring of the dynamic interactions between *H. pylori* and immune cells (31, 32). This system more accurately reflects the true infection scenario in humans and provides a new platform for studying the interactions between *H. pylori* and the host immune system.

Despite decades of immunological research, the development of immunotherapies for *H. pylori* infection (such as vaccines and immune checkpoint inhibitors) still faces many challenges (33). This is mainly due to the complex and diverse immune evasion mechanisms of *H. pylori*, which make it difficult for a single immunotherapy strategy to achieve satisfactory results. In addition, the immune response caused by *H. pylori* infection is highly heterogeneous, with significant differences in the immune responses of different individuals, which increases the difficulty of developing immunotherapies. At the same time, the current understanding of the immune regulatory mechanisms of *H. pylori* infection is not yet complete, and there is a lack of effective immune targets. Finally, limitations in the design of clinical trials are also a major issue. Existing studies have mostly focused on preventive vaccines for uninfected populations, while therapeutic vaccines for infected individuals have progressed slowly due to the need to break through the immune tolerance barrier. These factors together have led to the lag in the development of immunotherapies for *H. pylori* infection. Future research needs to further explore the immune regulatory mechanisms of *H. pylori* infection, identify effective immune targets, and develop personalized immunotherapies for *H. pylori* infection to improve therapeutic efficacy and ultimately reduce the disease burden associated with *H. pylori*.

## 3 Classification and functions of Th cells

**Figure 2:**
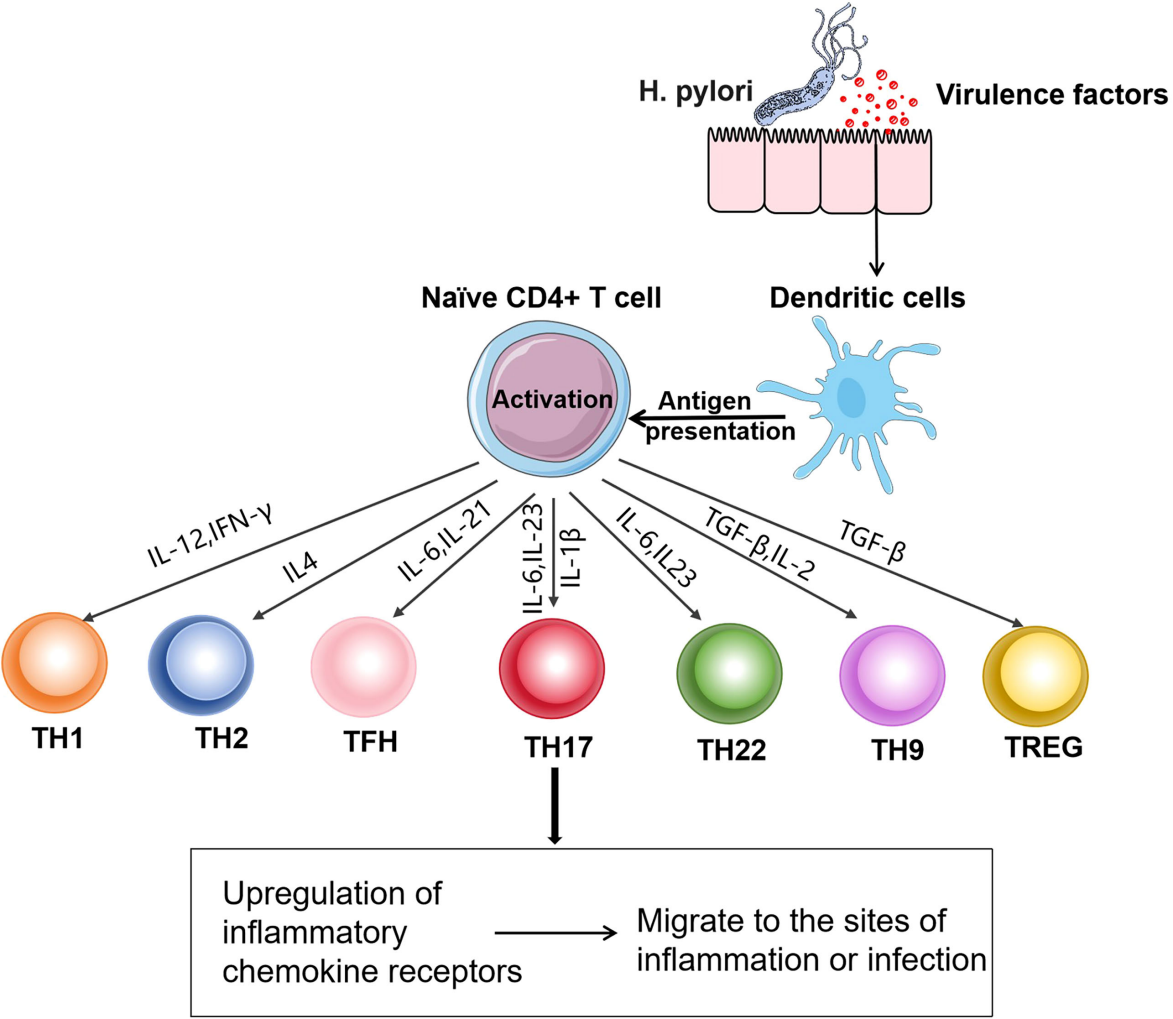
Differentiation of Th cell subsets: After *H. pylori* infection, dendritic cells present antigens to naïve CD4+ T cells. CD4+ T cells are activated after receiving the antigens presented by antigen-presenting cells. The activated CD4+ T cells differentiate into Th1, Th2, Tfh, Th17, Th22, Th9, and Treg cells under the action of different signals. These cells upregulate the corresponding chemokine receptors, enabling them to migrate from lymphoid tissues to the sites of infection or inflammation and exert functional effects.

T cells play a crucial role in maintaining good health and preventing diseases. They are the main cellular components of the adaptive immune system and are responsible for mediating cell-based immune responses to prevent the occurrence of various diseases (34). Based on the differences in T-cell receptors, T cells can be divided into αβT and γδT cells. Among them, αβT cells can be further classified into CD4+ T cells and CD8+ T cells according to the differences in cluster of differentiation (CD) on the cell surface. CD4+ T cells, also known as Th cells, when encountering microbial pathogens, antigen- presenting cells present antigens to naïve CD4+ T cells. Subsequently, naïve CD4+ T cells are activated. According to the cytokines they produce after activation and the immune effects they mediate, Th cells are divided into Th1, Th2, Th17, Th22, Th9, regulatory T cells (Tregs), and follicular helper T cells (Tfh) (35). These cells all have unique functional characteristics (36) (**Figure 2**), which are essential for host defense but are also the main drivers of immune-mediated diseases (**Table 1**).

**Table 1:** Helper T cell subsets and their functions.

Subsets of helper T cells	Cytokines required for activation	Transcription factor	Secretory factor	Functions	References
Th1	IL-12, IFN-γ	Tbet	IFN-γ,IL2,TNF-α	Mediate macrophage activation, kill intracellular pathogens and also induce tissue inflammatory damage	(37)
Th2	IL-4	GATA3	IL3,IL4,IL5,IL6, IL10	Mitigated the inflammatory damage of the gastric mucosa, but also facilitated the chronic progression of *H. pylori* infection by enabling the pathogen to evade host immune clearance	(38, 39)
Tfh	IL-6,IL-21	Bcl6	IL6,IL10,IL21	Stimulate the proliferation and differentiation of B cells in germinal centers	(40)
Th17	IL-6,IL-23,IL-1β	RORγt	IL17A,IL17F,IL21	Participate in the immune response to *H. pylori* infection	(41, 42)
Th22	IL-6,IL23	AHR,RORγt	IL22,IL23	It plays an important role in mucosal immunity and regulates the inflammatory response	(43, 44)
Th9	TGF-β,IL-2	DU.1	IL9	articipate in the immune response to *H. pylori* infection and exert certain immunological functions	(45)
Treg	TGF-β	Foxp3	IL10,TGF-β	Immunosuppression	(46)

In recent years, with the deepening of research on Th cells, it has been found that Th cells can exhibit different functional characteristics in various microenvironments. For instance, in the tumor microenvironment, certain Th cells may display inhibitory functions, suppressing the activity of other immune cells, thus promoting tumor growth and metastasis (47). Additionally, the metabolic state of Th cells is regarded as a crucial regulatory factor for their functions. Altering the metabolic pathways may affect their ability to secrete cytokines, thereby influencing the outcome of the immune response (48). In terms of clinical applications, treatment strategies targeting different subsets of Th cells are gradually evolving. For example, the inhibition of Th17 cells may contribute to the treatment of autoimmune diseases (49, 50), while enhancing the function of Th1 cells may improve the efficacy of cancer immunotherapy (51).

The development of single-cell sequencing technology has provided a new perspective for studying Th cell subsets in *H. pylori* infection. Chiara Sorini and colleagues performed single-cell sequencing on gastric tissues from *H. pylori*-infected patients and uninfected individuals, expanding the current understanding of immune cell functions in both uninfected and *H. pylori*-infected gastric mucosa. This may help develop new therapeutic strategies targeting Th cells to treat less benign gastric infections and their severe consequences (52). However, traditional single-cell RNA sequencing, although capable of revealing cell type composition, struggles to capture spatial interactions within the tissue microenvironment. Spatial transcriptomics, an emerging high-throughput technology, can provide information on the spatial location and gene expression of cells within tissues. This technology is particularly important for studying the distribution and function of T cells in tissues. If spatial transcriptomics could be used to elucidate the spatial distribution of T cells in the gastric mucosa and their interactions with surrounding cells, researchers would gain a more comprehensive understanding of the mechanisms by which T cell subsets contribute to infection and inflammation.

## 4 The role of Th cells in *H. pylori* infection

*H. pylori* is a bacterium that widely colonizes the human gastric mucosa. It can evade the host’s immune response and persist for a long time. Th cells play a significant role in the immune response to *H. pylori* infection, yet they also contribute to mucosal inflammation. Research indicates that Th cells are not only involved in the control of *H. pylori* but may also promote the development of mucosal inflammation (53).

### 4.1 Th1 and *H. pylori* infection

**Figure 3:**
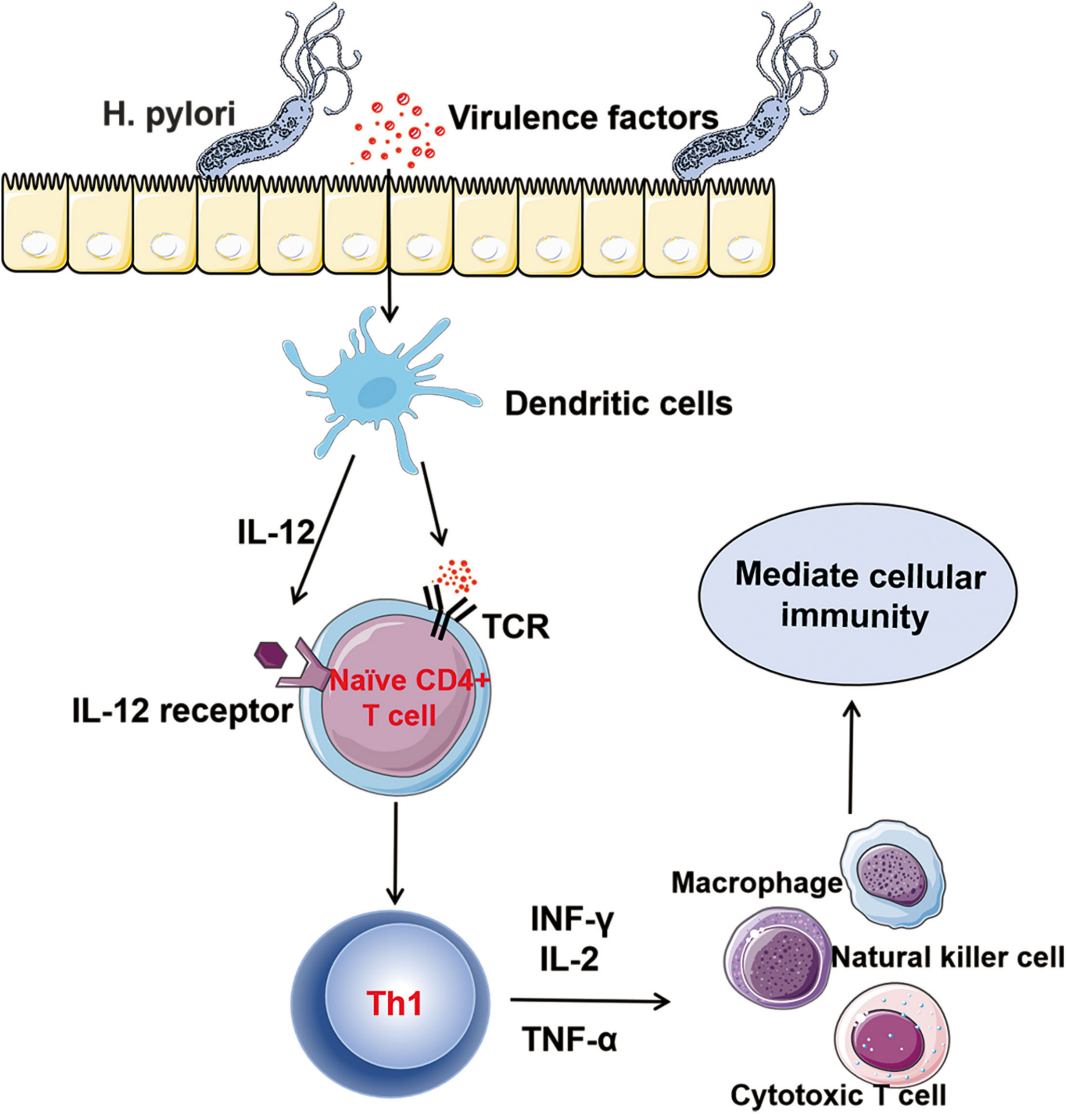
Th1 and *Helicobacter pylori* Infection: After being infected with *Helicobacter pylori*, the virulence factors secreted by *Helicobacter pylori* stimulate dendritic cells to produce IL-12 and present antigens to naïve CD4+ T cells, promoting the differentiation of TH0 cells into TH1 cells. Th1 cells mainly secrete cytokines such as INF-γ, IL-2, and TNF-α. They mediate cellular immunity by promoting the activation and proliferation of natural killer (NK) cells, cytotoxic T cells, and macrophages.

Th1 cells are one of the first - discovered subsets of Th cells. In the early stages of *H. pylori* infection, the Th cell - related immune response triggered by *H. pylori* is strongly skewed towards the Th1 type. The virulence factors secreted by the bacteria stimulate dendritic cells to produce IL - 12 and present antigens to naïve CD4^+^ T cells, promoting the differentiation of Th0 into Th1 cells (54, 55). The differentiation process of Th1 cells involves the activation of the key transcription factor T-bet, which in turn regulates the expression of cytokines such as IFN-γ, thereby enhancing Th1 polarization (56). The Th1 cell subset mainly secretes cytokines such as IFN-γ, IL-2 and IL-12 (57, 58). These factors mediate the cellular immune process by promoting the activation and proliferation of natural killer cells, cytotoxic T cells and macrophages (37) (**Figure 3**). During *H. pylori* infection, the activation of macrophages by the cytokine IFN-γis highly emphasized. IFN-γ can bind to receptors on the surface of macrophages, activating the signaling pathways within macrophages. This prompts macrophages to express more phagocytosis - related receptors, such as scavenger receptors and Fc receptors, thereby enhancing the recognition and uptake efficiency of *H. pylori* by macrophages and strengthening their ability to phagocytose and kill *H. pylori*. IFN-γ can also induce macrophages to produce bactericidal substances such as nitric oxide and reactive oxygen species, enhancing their killing effect on *H. pylori* (59, 60). Lewis N. D et al. found that after co - culturing macrophages infected with *H. pylori* with IFN-γ, the activity of inducible nitric oxide synthase in macrophages increased significantly, the production of nitric oxide rose markedly, and the number of *H. pylori* decreased significantly (61). This fully demonstrates that IFN-γ can effectively enhance the killing ability of macrophages against *H. pylori* by activating them. However, the immune response of Th1 to *H. pylori* is largely unable to completely and effectively clear the infection. As the colonization amount of *H. pylori* gradually increases, the production of cytokines that promote the differentiation of Th1 in the stomach increases, and Th1 cells are continuously activated. The persistent excessive Th1 cell-related reactions may lead to damage to the gastric mucosal tissue and contribute to the chronicization and exacerbation of gastric mucosal inflammation. The long-term excessive secretion of IFN-γ can induce gastric mucosal epithelial cells to produce chemokines, which recruit a large number of inflammatory cells to infiltrate, forming chronic inflammatory lesions and subsequently leading to the occurrence of gastritis and peptic ulcers (62). In addition, during the chronic infection stage, the activation of cyclooxygenase (COX)-2 in the gastric mucosa infected with *Helicobacter pylori* increases. It inhibits the Th1 induction against *Helicobacter pylori*, causing the Th1/Th2 balance to tilt towards Th2, which is not conducive to the clearance of the pathogen (63, 64). Overall, the role of *H. pylori* antigens in the nature of the Th1 response remains controversial. Future studies that can clarify this controversy will be helpful for the development of new treatment regimens targeting the immune response.

### 4.2 Th2 and *H. pylori* infection

Under normal physiological conditions, Th1/Th2 cells are in a dynamic equilibrium, jointly maintaining the body’s immune homeostasis (65). Th1 cells mainly mediate cell - mediated immunity and play an immunodefensive role against intracellular pathogen infections. In contrast, Th2 cells mainly mediate humoral immunity and function against extracellular pathogen infections and in allergic reactions (66, 67). During the early stage of *H. pylori* infection, the Th2 cell - mediated immune response is relatively weak, and the body mainly relies on the Th1 mediated cell - mediated immune response to combat the infection (68). At this stage, if the body’s immune function is strong enough to effectively eliminate *H. pylori*, patients may only experience mild gastric discomfort symptoms, such as transient dyspepsia and mild upper abdominal pain. The disease progresses relatively slowly, and there is even a possibility of spontaneous recovery. However, if *H. pylori* is not cleared in a timely manner and the infection enters the persistent infection stage, Th2 cells can become overactivated. The differentiation of naïve CD4 T lymphocytes into Th2 cells is induced by interleukin - 4. After IL - 4 binds to the relevant receptors on T cells, it signals through Signal Transducer and Activator of Transcription 6 (STAT - 6), leading to the expression of GATA - Binding Protein 3 (GATA - 3), a Th2 - specific master transcription factor. GATA - 3 induces the release of Th2 - characteristic cytokines, including IL - 4, IL - 5, and IL - 13 (69). Although Th2 - related immune responses can defend against extracellular bacteria, Th2 - related responses specific to *H. pylori* are relatively weak (70). It has been reported that infected individuals develop local and systemic *H. pylori* - specific antibodies, which are considered to play a minor role in immune protection against the bacteria (38).

In *H. pylori* - associated gastric cancer, genetic polymorphisms of the T - bet gene and overexpression of GATA - 3 in T cells have been documented, indicating that an imbalanced shift from Th1 to Th2 responses during infection progression may lead to adverse outcomes in gastric diseases (39). This implies that in the chronic phase of *H. pylori* infection, enhanced Th2 immune responses may suppress Th1 cell function and disrupt the balance between Th1 and Th2 cells. This state of immune imbalance may weaken the body’s ability to clear *H. pylori*, allowing *H. pylori* to survive and multiply continuously in the stomach, thus accelerating the progression of the disease and leading to poor prognosis of gastric lesions (39). For example, in the development of mucosa - associated lymphoid tissue lymphoma, the abnormal activation of the Th2 cell - mediated immune response is considered to play an important role (71). The continuous immune response triggered by *H. pylori* infection can lead to the aggregation and proliferation of lymphocytes in the local gastric mucosa. The cytokines secreted by Th2 cells may promote the abnormal proliferation and differentiation of these lymphocytes, ultimately leading to mucosa-associated lymphoid tissue lymphoma. Moreover, changes in the Th2 cell-mediated immune response may also be associated with the development of gastric cancer (72). Long-term *H. pylori* infection causes repeated damage and repair of the gastric mucosa. During this process, the immune balance between Th1 and Th2 cells is disrupted, which may affect the proliferation, differentiation, and apoptosis of gastric mucosal epithelial cells, increasing the risk of cellular gene mutations and thereby promoting the development of gastric cancer. Therefore, modulating the Th1/Th2 balance to enhance the body’s ability to clear *H. pylori* will be a new therapeutic measure for *H. pylori* - related diseases.

### 4.3 Th17 cells and *H. pylori* infection

Th17 cells differentiate from naïve CD4+ T cells under the combined action of cytokines such as TGF-β, interleukin-6, interleukin-21 and interleukin-23 (73, 74). Retinoic acid-related orphan receptor γt (RORγt) is a key transcription factor for Th17 cell differentiation (75), It can regulate the expression of a series of genes related to Th17 cell differentiation and function, promoting the differentiation of naïve CD4+ T cells into Th17 cells. The main cytokines secreted by Th17 cells include interleukin-17, interleukin-21, IL-22 and interleukin-23 (76–78). Interleukin-17 is a signature cytokine secreted by Th17 cells. It can induce the production of antimicrobial peptides, epithelial cells, chemokines, cytokines, and metalloproteinases, and is capable of recruiting and activating neutrophils to participate in inflammatory responses (79, 80).

Previous studies have shown that *H. pylori* infection leads to a Th1 - dominated immune response. In recent years, it has been found that after *H. pylori* infection, the number of Th17 cells increases. In the early stages of infection, the induction of Th17 cell responses precedes that of Th1 cell responses, suggesting that Th17 and Th1 cells may promote gastric mucosal inflammation at different stages. Gil et al. reported that in children with *H. pylori* infection, the number of Th17 cells increased, and the number of Th17 cells was positively correlated with the degree of progressive acute inflammation, indicating that Th17 cells play an important role in the clearance of *H. pylori* (81). This process may be related to the release of IL - 17 by Th17 cells, which recruits and activates neutrophils to directly participate in the clearance of *H. pylori* (41). However, although the immune response mediated by Th17 cells plays an important role in the clearance of *H. pylori*, it may also promote the occurrence and development of *H. pylori* - related diseases to some extent. Related studies have shown that IL - 17 can further promote the production of IL - 8 by activating the ERK1/2 MAPK signaling pathway. The latter is believed to induce inflammatory responses, damage the gastric mucosa, and is closely related to tumors (42, 82). In addition, the Th17 response induced by *H. pylori* can activate Th1 immune responses that are conducive to *H. pylori* colonization (83, 84). At the same time, the cytokines, ROS, NO, and other substances released in this process further expand the inflammatory response and enhance oxidative stress, thereby exacerbating mucosal damage and epithelial cell apoptosis. Therefore, the immune response of Th17 cells also has a dual function after *H. pylori* infection.

### 4.4 Treg cells and *H. pylori* infection

Tregs are a subset of T cells with immunosuppressive functions, playing a crucial role in maintaining immune balance, preventing autoimmune diseases, and regulating inflammatory responses in the body. According to their origin and differentiation pathways, Tregs can be mainly divided into natural Tregs (nTregs) and induced Tregs (iTregs) (85). Natural Tregs mature in the thymus, accounting for approximately 5% - 10% of CD4+ T cells in the thymus. After recognizing self-antigens, they are retained through a negative selection process and then migrate to peripheral immune organs. Induced Tregs, on the other hand, differentiate from peripheral naïve CD4+ T cells under the combined action of specific antigen stimulation and a cytokine - rich environment. TGF-β plays a key role in the differentiation of induced Tregs (86), It can prompt naïve CD4+ T cells to express Foxp3, gradually transforming them into induced Tregs with immunosuppressive functions.

Tregs are one of the key mechanisms by which the body regulates inappropriate or excessive immune responses. They can suppress the strong immune reactions that the body generates after pathogen infection, thereby reducing tissue immune damage. However, they also protect the pathogens, leading to chronic infections (87). During acute *H. pylori* infection, the body needs a rapid and robust immune response to eradicate the pathogen. However, if eradication fails, a continuous strong but unsuccessful immune response can cause severe tissue damage. The mechanism by which acute *H. pylori* infection induces Tregs has not been fully elucidated to date. During chronic inflammatory states, several immune regulatory mechanisms are involved, one of which is the activation and expansion of iTregs. iTregs accumulate in the mucosal tissues of *H. pylori* - induced diseases and chronic infections (6). Recent studies have shown that Tregs suppress the immune response to *H. pylori* infection (46). The host’s failure to eradicate *H. pylori* may be due to the pathogen’s ability to evade T - cell immunity by inducing Tregs. Studies on mice infected with *H. pylori* have shown that Treg depletion leads to increased gastritis and reduced colonization of *H. pylori* infection. These findings suggest that Tregs contribute to the persistence of *H. pylori* colonization in the gastric mucosa, leading to chronic infection.

### 4.5 *H. pylori* promotes immune evasion by disrupting the Th17/Treg balance

Th17 cells accumulate in the inflamed stomach and produce pro - inflammatory cytokines such as IL - 17A, IL - 17F, and IL - 21 (88). In contrast, Treg cells effectively suppress pathogenic T - cell responses through IL - 10 and TGFβ (89). Therefore, the Th17/Treg balance usually reflects the balance between pro - inflammatory and anti - inflammatory effects, controlling gastrointestinal mucosal immune homeostasis. Over - activation of Th17 cells or abnormal increase of Treg cells can break this balance and lead to disease progression. Studies have shown that *H. pylori* infection can up - regulate the expression of the T - cell co - inhibitory molecule B7 - H1 while down - regulating the expression of B7 - H2. This change can affect the balance between Treg and Th17 cells, thereby promoting bacterial persistent infection (90). It has been found that in children infected with *H. pylori*, the number of Treg cells and Th17 cells both increased, but the Th17/Treg balance shifted towards Treg. Treg cells suppress immune responses, which is conducive to *H. pylori* immune evasion and forms a long - term inflammatory stimulus (81). *H. pylori* can actively shift T - cell responses towards a regulatory phenotype, thereby inhibiting Th17 - driven immunity and promoting persistence. The proposed mechanisms involve the interaction between *H. pylori* and dendritic cells. When exposed to bacteria *in vitro*, dendritic cells seem to preferentially induce regulatory T cells rather than Th17 responses and are unable to produce pro - inflammatory cytokines (91, 92). This indicates that after *H. pylori* infection, by inhibiting DC cell maturation or reprogramming them, the Th17/Treg balance is shifted towards Treg. Although the number of Th17 cells also increases, the final outcome is Treg - dominant immune evasion, leading to chronic and persistent infection. In fact, in some infected individuals, chronic infection can lead to atrophic gastritis and gastric adenocarcinoma (93). Therefore, after *H. pylori* infection, how to regulate the Th17/Treg balance in the body is a breakthrough for the treatment of related diseases.

### 4.6 Th22 cells and *H. pylori* infection

Th22 cells are a subset of Th cells discovered in recent years. They differentiate from naïve CD4+ T cells under the combined action of cytokines such as TNF-α, interleukin-6, and interleukin-23. The hallmark cytokine of Th22 cells is IL-22. In addition, they also secrete cytokines such as interleukin-13 and TNF-α (94). The aryl hydrocarbon receptor (AHR) is a key transcription factor for Th22 cells. Upregulation of AHR expression can promote the secretion of IL - 22 and reduce the production of IL - 17, which is important for maintaining the function of Th22 cells (95). Th22 cells play an important role in skin and mucosal immunity. They can promote the proliferation and repair of epithelial cells and enhance the mucosal barrier function (43). Moreover, Th22 cells can regulate the inflammatory response and play a dual role in different types of infections (44). In infections caused by influenza viruses (96), Th22 cells and IL-22 enhance the host’s defense ability. However, in hepatitis B (97) and persistent fungal infections, Th22 cells and IL-22 increase tissue damage and degradation by exacerbating inflammation. When the gastric mucosa is infected with *H. pylori*, Th22 cells may be activated and secrete IL-22. IL-22 binds to IL-22R on the surface of gastric mucosal epithelial cells, activating the intracellular signaling pathway, promoting the proliferation and repair of gastric mucosal epithelial cells, and enhancing the barrier function of the gastric mucosa to resist further invasion by *H. pylori*. However, if the immune response of Th22 cells is excessive or dysregulated, it may lead to the exacerbation of the inflammatory response and cause damage to the gastric mucosa (98). Studies have shown that the number of Th22 cells in the gastric mucosal tissue of patients infected with *H. pylori* is significantly higher than that in healthy individuals. In a study on patients with different gastric diseases accompanied by *H. pylori* infection, it was found that the degree of Th22 cell infiltration in the lamina propria of the gastric mucosa of gastritis patients was significantly higher than that in the normal control group, and the number of Th22 cells increased with the aggravation of gastritis (98, 99). This indicates that Th22 cells may be involved in the gastric inflammatory response process triggered by *H. pylori* infection, and the change in their number is closely related to the severity of inflammation.

### 4.7 Th9 and *H. pylori* infection

Th9 cells were initially considered a subset of Th2 cells. Subsequent studies revealed that Th9 cells do not secrete the Th2 - characteristic cytokines IL - 4, IL - 5, and IL - 13. Therefore, Th9 cells are now regarded as a new subset of CD4+ T cells. They differentiate under the combined induction of IL - 4 and TGF -βby activating various downstream transcription factors such as purine - rich box 1 (PU.1), interferon regulatory factor - 4 (IRF4), and signal transducer and activator of transcription - 6 (STAT6) (100, 101).

The characteristic cytokine of Th9 cells is interleukin-9 (102, 103), IL-9 is a cytokine with unique biological functions, and it exerts its biological effects by binding to the IL-9 receptor (IL-9R) on the surface of target cells. During the course of *H. pylori* infection, the number of Th9 cells and the expression level of IL-9 change significantly. Some studies have reported that the proportion of Th9 cells in patients with *H. pylori* - associated gastritis and peptic ulcer is significantly higher than that in healthy individuals, and the serum level of IL-9 also increases remarkably (45). This indicates that in gastritis induced by *H. pylori* infection, Th9 cells are activated and their numbers increase, accompanied by a corresponding increase in the secretion of their characteristic cytokine, IL-9. It has been reported in relevant studies that elevated levels of IL - 9 in human gastric mucosa are associated with *H. pylori* - positive status and gastric adenocarcinoma. Whether elevated IL - 9 levels can serve as a diagnostic marker for *H. pylori* - related diseases remains to be further investigated.

### 4.8 Tfh cells and *H. pylori* infection

The differentiation of Tfh cells is a multi - stage and multi - factor process (104). After the body is stimulated by an antigen, antigen - presenting cells present the antigen to naïve CD4+ T cells. Subsequently, the naïve CD4+ T cells are activated, marking the initiation of Tfh cell differentiation (105). During the first few rounds of cell division, CD4+ T cells make cell - fate decisions (106). Only cells that highly express the chemokine CXCR5 will migrate to the B - cell follicle and further differentiate into Tfh cells (107, 108). Tfh cells are mainly located in the lymphoid follicles of peripheral immune organs. In the lymphoid follicles, Tfh cells can closely interact with B cells, forming a unique intercellular interaction network. The interaction between Tfh cells and B cells is particularly significant in the germinal center, a special structure within the lymphoid follicle. Research reports that *H. pylori* infection can activate the NF - κB signaling pathway in host cells, thereby promoting the differentiation and enhanced function of Tfh cells (40). In addition, *H. pylori* further promotes the functions of Tfh cells and the activation of B cells by upregulating cytokines such as IL - 21. These cytokines are crucial for antibody production, as they can promote the transformation of B cells into plasma cells, thus enhancing antibody secretion. They play an important role in the initial immune defense of the body against *H. pylori* infection (109).

In summary, during the course of *H. pylori* infection, different subsets of helper T cells play unique roles. Only when immune cells collaborate with each other and maintain a state of balance can the body’s immune equilibrium be sustained. This allows the body to effectively resist *H. pylori* infection while maintaining the relative stability of the gastric mucosa.

## 5 Conclusion

By integrating the functions and interactions of different helper T cell subsets, we have revealed the key roles of these cell subsets at different stages of *H. pylori* infection. However, currently, our understanding of the roles and functions of T - lymphocyte subsets in combating *H. pylori* infection is limited. Th1 and Th17 cells appear to be involved in the pro - inflammatory activities induced by this bacterium, but neither subset alone can automatically clear the infection. Th2 cells can reduce bacterial load, yet their protective role remains controversial. The fundamental role of limiting local inflammation and tissue damage is associated with the Treg cell pathway, but their functions may be exploited by *H. pylori*, leading to persistent bacterial infection. Therefore, further exploration is needed to fill the existing knowledge gaps. Most of the current immune - cell - based treatment strategies are at the laboratory research stage, and there is still a significant gap before they can be applied in clinical practice. How to precisely regulate the functions and quantities of these immune cells while avoiding severe immune - related adverse reactions is an urgent problem to be solved. Additionally, existing research mostly focuses on the analysis of single immune cells, lacking a systematic study of the combined effects of multiple immune cells. At present, cutting - edge technologies such as single-cell sequencing and gene editing are developing rapidly. In the future, our research direction could focus on using these technologies to deeply analyze the functional changes and inter - regulatory mechanisms of various immune cells at different stages of *H. pylori* infection. For example, single - cell sequencing can be used to reveal the transcriptomic information of individual cells and precisely depict the dynamic changes of immune cell subsets. Multi - omics technologies can provide a comprehensive perspective to help understand the interactions and functional regulation between immune cells. At the same time, biomarkers are also important in the diagnosis and treatment of diseases. Understanding the changes in the proportions of T - cell subsets, cytokine levels, and T - cell exhaustion markers during the *H. pylori* infection process can help with early disease diagnosis and disease monitoring, and provide new directions for future therapeutic strategies, with the potential to improve the clinical treatment outcomes of *H. pylori* infection.
